# American Institute of Homœopathy

**Published:** 1894-12

**Authors:** George B. Peck

**Affiliations:** Providence, R. I.


### AMERICAN INSTITUTE OF HOMŒOPATHY.

### Bulletin No. 2.

The several members of the Executive Committee for 1895 having signified their approval, the next session will be held in the First Baptist Meeting-house, of Newport, R. I., commencing Thursday, June 20th, at 3 P. M., subject to a special contingency hereinafter to be indicated. On Friday evening, date subject to same contingency, a promenade concert and reception by the residents of Newport will be given at the Ocean House, from eight to eleven. The music will be furnished by D. W. Reeves' famous American Band. Attendants upon the Institute may find satisfactory accommodations in some one of the following hostelries:

The New Cliffs Hotel, Louis P. Roberts, of the Mitchell House, Thomasville, Ga., proprietor. Rates, $5.00 per day; special for Institute session, $4.50 per day. This is the only hotel overlooking the ocean; it also commands the bathing beach. It is the resort of the *crême de la crême* of Newport tourists. Though somewhat retired the electric cars, which pass within a few steps of its portals, place its guests in a few minutes at the doors of the First Church and in close proximity to all other important points. It can furnish one hundred persons with elegant accommodations. It will open June 14th.

The Ocean House, on Bellevue Avenue, with its two hundred and seventy-five rooms, or more, was conducted last year by Mr. Warren F. Leland. Who will have charge of it next year, and when it will open, I have not the slightest idea, but unquestionably some decent gentleman will hold its management. This uncertainty is somewhat bothersome to me, but need not trouble others. The headquarters of the Institute will be at this hotel. Regular rate, $5.00.

The Hotel Aquidneck, Thomas J. O'Neill, proprietor, has one hundred rooms, and can readily accommodate one hundred and fifty persons. It is the home of the Governor and Legislature the last week in May of each year. It is quiet, cozy, well-shaded, and perhaps seven minutes' walk from the Meeting-house. It is very centrally located, and its table is good. Regular rate $5.00, which may be shaded, say from $3.00 to $5.00, according to circumstances.

The Perry House, on Washington Square, is less than five minutes' distant from the First Baptist, which is, in a sense, in the rear of the State House. This is open the entire year, under the management of William S. O'Brian, and can receive, without difficulty, one hundred members. Its bill-of-fare is ample and satisfactory. The hotel is headquarters for commercial travelers, which fact is ample indorsement. Rate, $3.00 per day.

During the month of March, 1895, a list of suitable boarding-houses will be compiled, and applicants for rooms therein will be provided for in the order of the receipt of their requests, which may be sent at any time from now until June 8th, and should specify the size and character of the party, and the expected price. All communications should be addressed to the Secretary of the Local Committee of Arrangements,

George B. Peck, M. D.,
Providence, R. I.

